# Rehabilitation following hemorrhagic stroke: building the case for stroke-subtype specific recovery therapies

**DOI:** 10.12688/f1000research.11913.1

**Published:** 2017-11-23

**Authors:** Tomoko Kitago, Rajiv R. Ratan

**Affiliations:** 1 Department of Neurology, Columbia University, New York, USA; 2 Burke Medical Research Institute, White Plains, New York, USA; 3 Departments of Neurology and Neuroscience, Weill Cornell Medicine, New York, USA

**Keywords:** Rehabilitation, Hemorrhagic stroke, Recovery

## Abstract

Intracerebral hemorrhage (ICH), a form of brain bleeding and minor subtype of stroke, leads to significant mortality and long-term disability. There are currently no validated approaches to promote functional recovery after ICH. Research in stroke recovery and rehabilitation has largely focused on ischemic stroke, but given the stark differences in the pathophysiology between ischemic and hemorrhagic stroke, it is possible that strategies to rehabilitate the brain in distinct stroke subtypes will be different. Here, we review our current understanding of recovery after primary intracerebral hemorrhage with the intent to provide a framework to promote novel, stroke-subtype specific approaches.

## Introduction

Intracerebral hemorrhage (ICH) accounts for about 15% of all strokes in the USA and Europe and leads to significant mortality and long-term disability despite advances in acute medical and surgical treatment ^1, 2^. In humans, primary ICH occurs because of a rupture of an intracerebral vessel, most often due to hypertension or cerebral amyloid angiopathy. Vessel rupture leads to mechanical tissue disruption (secondary to the jet of blood) and hematoma formation, followed by secondary injury processes, including disruption of the blood-brain barrier, expansion of perihematomal edema, inflammation, neuronal cell death, and chemical or cellular barriers to repair ^3^. These secondary injury processes occur over hours to days after the primary injury and are attributable to toxins from lysed blood, including hemoglobin (Hgb), hemin (oxidized form of heme), and thrombi ^4– 8^. These toxins could affect cell survival or repair via interactions with a host of cell types, not only neurons.

Although research in the field of stroke recovery and rehabilitation has grown, there have been very few studies specifically focusing on rehabilitation in ICH. In this review, we summarize the recent advances in our knowledge of the pathophysiology of primary ICH, how rehabilitation affects functional outcomes after ICH, and the mechanisms that may underlie the gains seen with rehabilitation in preclinical models and patients.

## Pathophysiology of intracerebral hemorrhage

Preclinical models have provided important insights into the pathophysiological processes that occur after ICH, which differ from those seen after ischemic stroke ^9^. The two most commonly used rodent models of ICH are autologous blood injection and bacterial collagenase infusion ^10^. In the whole-blood injection model, lysed blood is injected directly into the brain parenchyma, with almost no spontaneous bleeding ^11^. The bacterial collagenase model involves intraparenchymal infusion of bacterial collagenase, an enzyme that damages the basal lamina and causes bleeding that evolves and grows over 24 hours ^12^. Models of hypertensive rodents in which ICH occurs spontaneously, which may more closely mimic what occurs in patients, have been less widely used. In reviewing the literature, one must consider that these preclinical models have different pathophysiological processes which may affect the course of recovery and response to rehabilitation; thus, the use of multiple models has been recommended to better understand post-ICH recovery in preclinical studies ^13^.

After ICH, there is both immediate and ongoing cell death in both whole-blood and collagenase models ^14^. In the collagenase model, there is markedly greater delayed cell death, leading to worse functional deficits even when the hematoma size is matched ^14^. Historically, it was believed that Hgb or hemin induced toxicity following ICH by loading neurons with redox-active free iron leading to the formation of hydroxyl radicals via Fenton chemistry ^9^. Indirect support for this model came from studies, not replicated by all groups, that iron chelators such as deferoxamine (DFO) improve functional recovery in pig ^15^ and rodent ^16^ models. More recent studies suggest that DFO and a more selective metal chelator, adaptaquin, act not by inhibiting Fenton chemistry but by inhibiting a specific family of iron-, 2-oxoglutarate-, and oxygen-dependent dioxygenases, the hypoxia-inducible factor proly hydroxylases (HIF PHDs) ^17^. HIF PHDs are oxygen sensors and accordingly, with drugs such as adaptaquin, the brain can be fooled into thinking it is hypoxic when it is not. The consequence of turning on hypoxic adaptation in ICH with adaptaquin is to inhibit the synthesis of a cassette of genes (Chac1, Trib3, and Xc-transporter) that are involved in mediating neuronal death. Accordingly iron, likely via its ability to act as a cofactor for the HIF PHDs, promotes the synthesis of ATF4-dependent genes that mediate neuronal death.

Of note, ATF4 regulates the synthesis of a pseudokinase inhibitor of AKT, called tribbles homolog 3 (Trib3). Studies in non-neural cells have shown that Trib3 and ATF4 can dimerize to inhibit transcription by the pro-plasticity transcription factor, CREB (cAMP-responsive element-binding protein). Activators of CREB have been shown to improve learning and memory as well as functional recovery after stroke. Accordingly, iron’s ability to load the HIF PHDs could lead to the transcriptional induction of genes (for example, Trib3) that constrain neuronal survival and plasticity following hemorrhagic stroke. The model is consistent with data showing that adaptaquin, a small-molecule inhibitor of the HIF PHDs, improves functional recovery out to a month in collagenase and blood infusion models in mice and rats, respectively. Future studies will clarify the role of ATF4 in regenerative failure following ICH.

How do cells die following ICH in cellular and rodent models? Recent studies from two groups suggest that cell death in ICH is completely distinct from that found following ischemic stroke. In ischemic stroke, cell death occurs via a poly(ADP-ribose) polymerase-1 (PARP)-dependent pathway leading to AIF release from the mitochondria and *Parthanatos* ^18, 19^. By contrast, recent data suggest that cell death following ICH occurs via a ferroptotic pathway that also involves necroptosis ^7, 20^. Of note, inhibitors of *Parthanatos* have no effect on ICH-induced death *in vitro* ^7^, suggesting that the pathways of cell damage and, by extension, regenerative failure might be different.

Other studies have focused on the role of the immune system in secondary injury and repair. After ICH, there is a marked inflammatory reaction with activation of microglia and recruitment of leukocytes to the perihematomal region, leading to the production of inflammatory mediators, including cytokines, chemokines, and matrix metalloproteinases ^9, 21^. Interestingly, activated microglia can take on pro-inflammatory (via classic activation) or anti-inflammatory (via alternative activation) phenotypes, the latter associated with wound healing and repair ^22, 23^. A recent study demonstrated that, after ICH, microglia have dynamic changes in gene expression that result in a transition from an early, pro-inflammatory phenotype to an anti-inflammatory phenotype during the resolution phase of ICH ^24^. Transforming growth factor-beta 1 (TGF-β1) was identified as the most likely mediator of this effect, and treatment with TGF-β1 improved functional outcomes in a murine model. Furthermore, early increases in TGF-β1 levels were also shown to be highly associated with improved 90-day outcomes in patients with ICH.

Another area of research involves the transcription factor peroxisome proliferator-activated receptor gamma (PPARγ), which plays a significant role in phagocytosis and modulating inflammation and neuroprotective mechanisms ^3^. In a preclinical study, treatment with a PPARγ agonist, rosiglitazone, promoted hematoma resolution, reduced neuronal damage, downregulated the expression of pro-inflammatory genes (tumor necrosis factor-alpha, interleukin-1beta, matrix metalloproteinase-9, and inducible nitric oxide synthase mRNA), reduced oxidative stress, and was associated with improved neurological function ^25^.

## Recovery after intracerebral hemorrhage

Post-stroke recovery has been widely studied, but the majority of research has focused only on ischemic stroke ^26– 29^. Given the differences in pathophysiology between ischemic stroke and ICH, one could assume that the recovery outcomes or mechanisms would be dissimilar between the two stroke subtypes. Comparisons between recovery outcomes in patients with ischemic stroke and ICH have yielded mixed results; some have found comparable activity limitation and recovery ^30^, whereas others have found greater recovery after ICH ^31^.

The majority of recovery after ICH occurs early, within the first few months post-stroke ^32^. A recent longitudinal study of patients with ICH characterized the time course of recovery of motor and sensory impairment and ambulation in 11 patients up to six months post-stroke ^33^. Sensory, truncal, and lower limb motor impairment reached a plateau after three months, whereas there was continued improvement of upper limb movement and ambulation to six months. The course of motor recovery has been shown to depend on the integrity of the corticospinal tract (CST), as measured by transcranial magnetic stimulation ^34^ or diffusion tensor tractography ^32^. In a study of 36 patients with putaminal hemorrhage, motor function recovered in all patients up to four months post-stroke, but patients who had preserved CST integrity at baseline had greater recovery of motor function ^32^. More studies are needed to better elucidate the natural history of recovery after ICH, as has been done for ischemic stroke.

## Preclinical studies of rehabilitation after intracerebral hemorrhage

Rehabilitation has been shown to lead to functional improvements in rodent models using both collagenase-induced and whole blood-induced ICH ^13, 35, 36^. Different types of rehabilitation have been tested in preclinical models, including environmental enrichment ^37, 38^, skilled reach training ^37^, constraint-induced movement therapy ^39, 40^, and aerobic training ^41^. Enriched rehabilitation (ER) combines environmental enrichment (group housing and access to tunnels, ramps, and various toys) and task-specific training (most commonly skilled reach training) and was first used as a successful rehabilitative intervention in preclinical models of ischemic stroke ^42, 43^. Rehabilitation has been shown to be associated with improved behavioral recovery and enhanced neuroplasticity, including dendritic reorganization ^44^, astrocytic plasticity ^45^, and synaptogenesis in ipsilesional motor cortex and striatum ^46^.

In a collagenase-induced rodent model, rats that participated in a two-week course of enriched rehabilitation beginning one week after ICH improved their skilled reaching and walking ability ^36^. ER also significantly reduced the amount of perihematomal neuronal death even after one week of treatment, suggesting a possible neuroprotective effect of rehabilitation. This study also examined whether rehabilitation influenced iron toxicity and inflammation post-ICH; rehabilitation did not have an effect on levels of iron-binding proteins (ferritin and transferrin) or number of inflammatory cells in perihematomal tissue. The results of a follow-up study also support a neuroprotective role of rehabilitation; ER beginning one week after ICH was demonstrated to augment the clearance of toxic blood components, Hgb and iron, and reduce oxidative stress at the hematoma/perihematomal interface ^35^. The authors speculate that rehabilitation upregulates pathways for Hgb clearance (for example, Nrf2 ^47^) and downregulates anticlearance pathways (for example, CD47 ^48^) to expedite hematoma resolution and limit secondary injury. Further investigation is needed into a possible neuroprotective role of rehabilitation in preclinical models, as well as the extent to which secondary injury processes contribute to the functional deficits after ICH in humans, and the ability to reduce secondary injury with rehabilitation in humans.

In a whole-blood injection model, ER beginning one week after ICH also led to significant improvements in reaching ability ^13^. Interestingly, the behavioral improvements in the whole-blood model, unlike those in the collagenase model, were not accompanied by a decrease in lesion volume or increase in dendritic length ^13^. The authors propose that the lack of delayed injury in the whole-blood model compared with the collagenase model may account for these differences. It is still unknown which of these models better exemplifies what occurs after ICH in humans.

Taken together, evidence from preclinical studies shows a benefit of early rehabilitation after ICH, but the mechanisms underlying the behavioral gains are incompletely understood and vary according to model. Further refinement of neurorehabilitative interventions in preclinical models, which focuses on factors such as the timing, intensity, schedule, and total dose of therapy, is also needed to guide the development of optimal rehabilitation paradigms for patients ^49^.

Rehabilitation may be more effective after hemorrhagic stroke than after ischemic stroke, when matched for baseline clinical severity. In a preclinical study in size- and location-matched ischemic or hemorrhagic stroke, rodents with ICH were shown to have greater recovery of skilled walking ability than those with ischemic stroke ^50^. Similar results were seen in ICH patients who received the same type and dose of inpatient rehabilitation and had greater recovery of neurological impairment and reduced activity limitation by time of discharge, relative to patients with ischemic stroke ^31^.

## Clinical rehabilitation after intracerebral hemorrhage

There have been very few rehabilitation studies undertaken in patients with ICH. Many of the larger neurorehabilitation trials have included both hemorrhagic and ischemic stroke patients ^51, 52^ or excluded ICH patients altogether from study ^53^. In animal models of ischemic stroke, there is an early ‘sensitive period’ after stroke, during which rehabilitation leads to larger behavioral improvements than when rehabilitation is delayed, even by one week ^42, 54^. However, owing to the paucity of clinical trials, evidence is lacking for a ‘sensitive period’ in humans for ischemic or hemorrhagic stroke.

The effects of early rehabilitation after ICH were recently investigated in a multicenter, prospective, randomized controlled study of 243 patients with ICH in China ^55^. Patients with moderate motor impairment were randomly assigned to either very early rehabilitation (within 48 hours) or standard care (rehabilitation starting after seven days). The very early rehabilitation group had shorter lengths of stay, improved quality of life, greater independence in activities of daily living, and lower six-month mortality when compared with the standard-care group. Because this study was conducted in China, where rehabilitation is delivered by family members and is less standardized, it is unclear whether the results are generalizable to the general ICH population. Furthermore, information on known predictors of outcome, such as hematoma volume, was not available, and the outcomes were self-reported and subject to responder bias. Nevertheless, it is a promising result in favor of very early rehabilitation, which has been a topic of recent debate ^56^.

Most clinical guidelines support early initiation of rehabilitation after stroke ^57^, and multiple studies have shown that early therapy is safe and feasible ^58, 59^. However, the results of the recently published A Very Early Rehabilitation Trial (AVERT) indicate that very early, intensive mobilization may be detrimental after stroke ^52^. In this single-blinded, randomized controlled trial of 2,104 patients with ischemic or hemorrhagic stroke, patients who received very early mobilization (< 24 hours post-stroke with more frequent sessions) were more likely to have an unfavorable outcome than patients who received usual care ^52^. In a pre-specified subgroup analysis, this effect was stronger in patients with severe stroke and ICH. Furthermore, providing early rehabilitation for patients with ICH is particularly challenging because these patients are often admitted to intensive care units and require close neurological and hemodynamic monitoring. One must ensure that rehabilitation interventions do not cause detrimental fluctuations in blood pressure and intracranial pressure that can lead to hematoma expansion, which is associated with worsened functional outcomes after ICH ^9, 60^.

## Conclusions and future directions

Reducing impairment and disability after ICH requires a multifaceted approach with advances in the acute medical and surgical treatment and rehabilitation of patients with ICH. Rehabilitation leads to significant behavioral improvements in animal models of ICH and is associated with enhanced neuroplasticity and reduced neuronal degeneration, although the mechanisms contributing to behavioral recovery are not yet understood. Clinical rehabilitation studies in patients with ICH have been limited; more studies are needed to characterize the natural history of recovery after ICH and to examine the effect of rehabilitative interventions specifically in this patient population. Therapies to enhance neuroprotection and neuroplasticity are also being developed and may be used in conjunction with rehabilitation to promote recovery.
